# Assessment of mismatch repair proteins (MLH1 and MSH2) and p53 immunohistochemical expression in prostatic carcinoma: association with different clinicopathologic characteristics

**DOI:** 10.3332/ecancer.2025.1985

**Published:** 2025-09-04

**Authors:** Hend S Abo Safia, Ahmed F Ghaith, Eman E Farghal, Basma S Amer

**Affiliations:** 1 Pathology Department, Faculty of Medicine, Tanta University, El Geesh Street, Tanta 31527, Egypt; 2 Department of Basic Medical Sciences, Faculty of Medicine, Ibn Sina University for Medical Sciences, Amman 16197, Jordan; 3 Department of Urology, Tanta University, El Geesh Street, Tanta 31527 Egypt; 4 Department of Clinical and Chemical Pathology, Faculty of Medicine, Tanta University, El Geesh Street, Tanta 31527, Egypt; a http://orcid.org/0000-0001-9117-4441; b http://orchid.org/0000-0002-9102-1821; c http://orcid.org/0000-0002-7562-055X; d http://orchid.org/0000-0003-0123-3255

**Keywords:** prostate cancer, mismatch repair proteins MSH2, MLH1, P53

## Abstract

**Background:**

Prostate cancer (PCa) is one of the most heritable human cancers and it is the second most frequent malignancy in men worldwide. It accounts for a significant morbidity and mortality throughout the world. PCa with mismatch repair (MMR) deficiency often has aggressive clinical and histological features, but its rarity prevents the analysis of the underlying biology. Therefore, in this study, we aimed to evaluate the immunohistochemical expression of MMR proteins and P53 in PCa.

**Materials and methods:**

Fifty cases of PCa were histologically examined. The MMR proteins and P53 immunoexpression were assessed. Also, P53 serum concentration levels using ELIZA was measured and pre-operative prostatic specific antigen (PSA) serum levels were obtained.

**Results:**

There was a significant positive relation between mutS homologue 2 (MSH2) immunoexpression and both PSA serum level and P53 serum concentration (*p* value 0.001*). Also, there was a significant relation between MSH2 immunoexpression and tumour size, nodal metastasis, distant metastasis and grade grouping. While mutL homologue 1 (MLH1) immunoexpression showed a significant relation with human P53 serum concentrations only (*p* value 0.035*). Moreover, MLH1 immunoexpression showed only significant relation with nodal metastasis and tumour burden, *p* value was 0.033* and 0.001*, respectively.

**Conclusion:**

MMR protein loss, especially MSH2, was seen in a significant subset of PCa. Interestingly, it was associated with significantly higher levels of serum PSA and p53. Moreover, it may be associated with unfortunate prognostic features as large tumour size, higher grade grouping and finally nodal and distant metastasis.

## Background

Prostate cancer (PCa) is considered the second most common cancer and the fifth leading cause of cancer mortality among aging men worldwide. There is an urgent need for clinically useful biomarkers for early detection of PCa to lessen the liabilities of overtreatment and accompanying tumour morbidity [25].

There are many risk factors for the development of PCa, including age, ethnicity and genetic factors. PCa is considered one of the most heritable human cancers and its heritability was estimated to be 57% in some populations, which is higher than the rates for almost all other cancers, including breast, kidney and ovarian cancer (31%, 38% and 39%, respectively) [17].

The typical morphology of prostate carcinoma is 'small gland' which has rigid round lumens, and often has characteristic infiltrative growth pattern. But the 'large gland' morphology is not infrequently detected [16]. They are large, crowded with irregular contour, papillary inholding and luminal undulation. Large gland prostate cancer may resemble adjacent benign glands architecturally, but are often larger than the latter [34]. Also, malignant glands are lined with cytologically atypical cells with nucleomegaly and prominent nucleoli [5].

The modifications of the International Society of Urological Pathology (ISUP) consensus conference in 2014 in Chicago were incorporated into the 2022 World Health Organization (WHO) Classification of Tumours of the Urinary System and Male Genital Organs. In the past 5 years, further new data in the Gleason pattern quantities, tumour growth patterns and clinical practice advancements such as widespread introduction of multiparametric magnetic resonance imaging-targeted biopsies or fusion ultrasound/magnetic resonance imaging biopsies have added to challenges in reporting and grading for pathologists [30].

The mismatch repair (MMR) deficiency causes functional abnormality of MMR proteins, resulting in their loss on immunohistochemistry (IHC). MMR is an excision-re synthesis system which acts as a sensor for DNA damage, correcting the mismatches generated during DNA replication. So, their deficiency leads to the accumulation of errors during DNA replication, especially in repetitive sequences known as microsatellites [19]. Defects in DNA MMR proteins are considered permissive for carcinogenesis, giving rise to microsatellite instability (MSI) and their role has been extensively studied in colorectal and endometrial cancer [1].

The recent studies on MMR protein expression in prostate tumours revealed that approximately 10% of advanced/metastatic prostate tumours have an underlying somatic and/or germline inactivation of genes in the MMR family (mutS homologue 2 (MSH2), MSH6, mutL homologue 1 (MLH1) or PMS2) [13,15]. This is similar to what has been detected in colorectal carcinoma [18, 24].

Many studies have searched for MMR defects in advanced PCa, but the relative frequency and clinical significance of MMR alterations in early PCa are less certain [6, 12].

As in many human cancers, p53 tumour suppressor gene mutations are a frequent genetic event in PCa, and can be detected in up to 94% of cases. With the exception of Li-Fraumeni syndrome and a very little fraction of other neoplasms in high-risk groups, most of the p53 abnormalities represent late somatic alterations in the process of carcinogenesis [3].

Many previous studies have focused on the role of p53 in prostatic cancer but most of them claimed that p53 gene mutations are not frequently detected in early-stage prostatic cancer. Recently, increasing evidence suggests that loss of p53 function may be an important early step in disease progression. Moreover, clonally altered p53 in earlier stage disease may be a prognostic marker. In addition, several recent studies have shown frequent p53 alterations in hormone-refractory prostatic cancer [14, 29].

In this study, we evaluated the relationship between the immunohistochemical expression of MMR proteins MLH1, MSH2 and p53 in prostatic cancer and their relation to the available clinicopathological features.

## Methods

The current retrospective study included 50 cases diagnosed as prostatic cancer during the period from February 2021 to October 2023. The study was approved by Research Ethics Committee, Faculty of Medicine, Tanta University, with approval number (36150/12/22). Written informed consent forms were collected once all study participants received information about it. This retrospective study was conducted at the Urology together with Pathology and Clinical Pathology Departments of Tanta University Hospital. Serum levels of prostatic specific antigen (PSA) and P53 levels were also assessed.

All patients underwent digital rectal examination and 12 cores transrectal ultrasound-guided prostatic biopsy, which was done by an expert urologist. The patients diagnosed as prostatic adenocarcinoma were subjected to computerised tomography for abdomen and pelvis and bone scintigraphy for clinical staging.

All cases were subjected to routine haematoxylin and eosin staining and examined by two pathologists to confirm the diagnosis.

### Blood sampling

#### Determination of p53 and PSA parameters

For the patients who were diagnosed as prostatic adenocarcinoma, blood samples were collected under strict aseptic conditions into serum vacutainer tubes and approximately after 30 minutes tubes were centrifuged at 6,000 revolutions per minute for 10 minutes at room temperature to obtain serum for p53 and PSA analysis. Serum samples were stored at −80°C until analysis of the dependent variables. P53 and PSA concentrations were measured spectrophotometrically using a plate reader (Multiskan Spectrum, Thermo Lab Systems). A p53 enzyme-linked immunosorbent assay (ELISA) kit (Human p53 ELISA, Catalog No: BMS256; Bender MedSystems GmbH, Austria, Europe) was used to measure p53 protein concentration in serum samples according to the manufacturer’s protocol. Serum PSA was measured using ELISA kit (PSA ELISA Catalog Number SE120106) according to the manufacturer’s protocol. To eliminate inter-assay variance, all samples for a particular assay were thawed once and analysed in the same assay run.

### Histopathological evaluation

Sections from the studied cases were subjected to hematoxylin and eosin staining. Gleason score was assigned according to the 2022 WHO classification ISUP consensus ISUP [32].

### Immunohistochemical staining

Tumour sections (of 5 μm thickness) on positively charged slides were left to dry for 30 minutes at 37°C. Sections were then deparaffinised and antigen retrieval was performed in Dako PT link unit using high and low PH EnVision FLEX antigen retrieval solutions (reaching 97°C for 20 minutes). Immunostaining was achieved in Dako Autostainer Link 48. Briefly, peroxidase blocking reagent was applied, followed by incubation with primary antibodies for 30 minutes. After that horseradish peroxidase polymer was applied for 20 minutes and diaminobenzidine was applied as chromogen. The slides were then counterstained by hematoxylin.

The primary antibodies applied in this study were:

1- MLH1, a monoclonal antibody (mouse anti-hMLH1 antibody (clone ES05, 1:50; Dako, Glostrup, Denmark),
2- MSH2, a monoclonal mouse anti-hMSH2 antibody (clone FE11,1:50 Dako Glostrup, Denmark),
3- P53, a monoclonal antibody, clone DO-7; 1:50 Dako, USA).

### Interpretation immunohistochemical stains

#### Interpretation of MLH1, MSH2 immunostaining results

If the tumour showed total absence of nuclear staining with the adjacent normal tissue showing normal nuclear staining, the case was regarded as deficient MMR. While tumours with normal nuclear staining in the neoplastic cells were regarded as having proficient MMR [8, 9].

#### Interpretation of P53 immunostaining results

Immunoreactivity was regarded as positive when brown staining was localised to the nucleus of the neoplastic cells. The evaluation of the IHC slides was done semi-quantitatively, and the staining was scored based on intensity as follows: 0, negative staining; 1+, weak staining; 2+, moderate staining and 3+, strong staining. For the statistical analysis, the cases were categorised in two groups, 0 and 1+ as low expression group and +2 and +3 as high expression group. The adjacent benign glands should not show more than weak, partial staining, if any. Negative staining pertains to no staining or focal, weak fine granular staining [21].

Positive controls for p53 nuclear accumulation used with each run of staining were a colon cancer specimen with p53 missense mutation.

### Statistical analysis

Data were statistically analysed using Statistical Package for the Social Sciences (version 23.0). Data were expressed as frequencies for categorial data, while mean ± SD was used to represent numerical data. Chi square test was used to compare categorial data, while Fischer exact or Monte-Carlo tests were used when appropriate. Student *t*-test was used to compare numerical data. *p* values of less than 0.05 were considered statistically significant.

## Results

### Clinicopathological features

The present study included 50 cases of prostatic adenocarcinoma. Patient characteristics were shown in Table 1. The age of the patients ranged from 52 to 87 years old. PSA plasma levels ranged from 0.50 to 100 ng/dL. Human p53 serum concentration levels ranged from 0.78 to 50 U/mL.

### Clinical staging

According to Table 1, 64% of cases were T1c and T2 equally, while 22% of cases were T4 and only 14% of cases were T3. Lymph node (LN) metastasis was found in 44% of cases. Usually, LNs with a short axis >8 mm in the pelvis and >10 mm outside the pelvis are considered malignant. Distant metastasis was only present in 14% of cases at the time of diagnosis.

### Pathological findings

Regarding perineural invasion, it was detected in 44% of cases. Gleason score was defined and most of the studied cases (40%) were well differentiated prostatic adenocarcinoma, while moderately differentiated PCa and poorly differentiated PCa represented in 28% and 32% of studied cases, respectively, as shown in Table 1.

**Figure 1.**
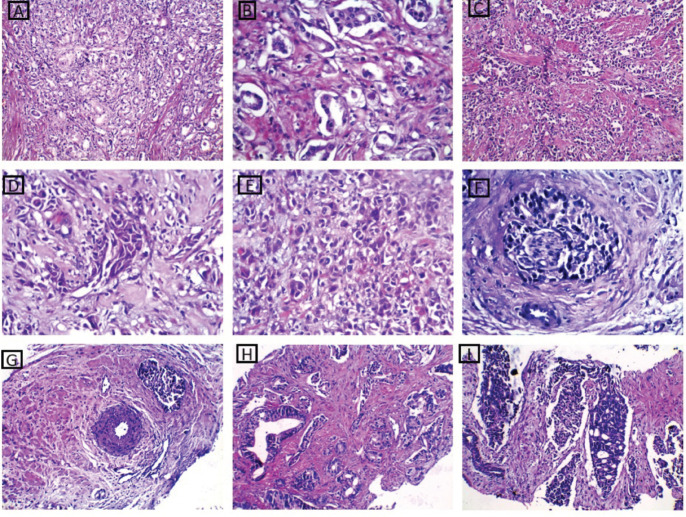
H&E-stained sections from studied PC cases. (a and b): A case of well-differentiated PC Gleason score 3 showing uniform glands with spaces between them. The glands are lined by a single layer of malignant cells (×200 and ×400, respectively. (c–e): Cases of poorly differentiated PC Gleason score 9 showing infiltrating fused glands admixed with sheets and cords of malignant cells ×200 and ×400, respectively. (f): A case of moderately differentiated PC Gleason score 7 showing perineural infiltration ×400. (g): a case of moderately differentiated PC Gleason score 7 showing malignant glands near muscular blood vessel ×400. (h and i): Moderately differentiated PC Gleason score 7 showing medium sized glands with undulating luminal contours and large branching glands with areas of cribriforming ×200.

After careful histopathological examination (Figure 1) of all cases and defining the exact Gleason score for each case, Grade Grouping was done and 54% of cases were Grade Group 1, while Grade Group 2 was the least represented in the current study, 6% of cases. Grade Groups IV and V represented 14% and 18%, respectively.

### MMR proteins immunoexpression results

**Figure 2.**
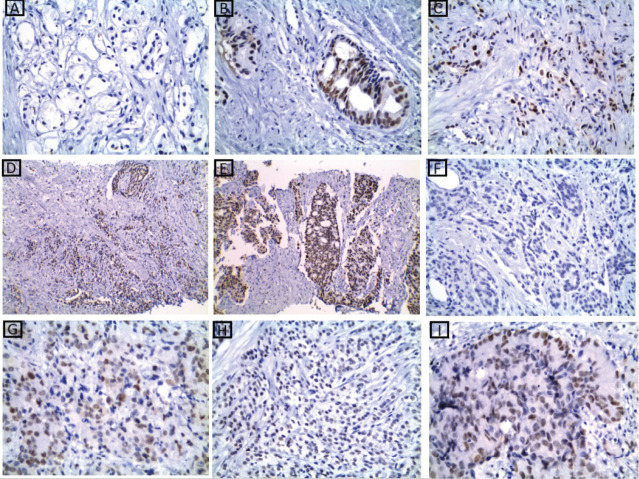
MSH2 immunohistochemical results. (a): A case of well-differentiated PC Gleason score 3 showing absent nuclear expression of MSH2 ×200. (b and c): Two cases of moderately differentiated PC Gleason score 7 showing retained nuclear expression of MSH2 ×400. (d): A case of poorly differentiated PC Gleason score 9 showing retained nuclear expression of MSH2 ×200. (e): A case of moderately differentiated PC Gleason score 7 showing retained nuclear expression of MSH2 ×200. (f): A case of moderately differentiated PC Gleason score 7 showing absent nuclear expression of MSH2 ×400. (g–i): cases of poorly differentiated PC Gleason score 9 and 10 showing retained nuclear expression of MSH2 ×400.

**Figure 3.**
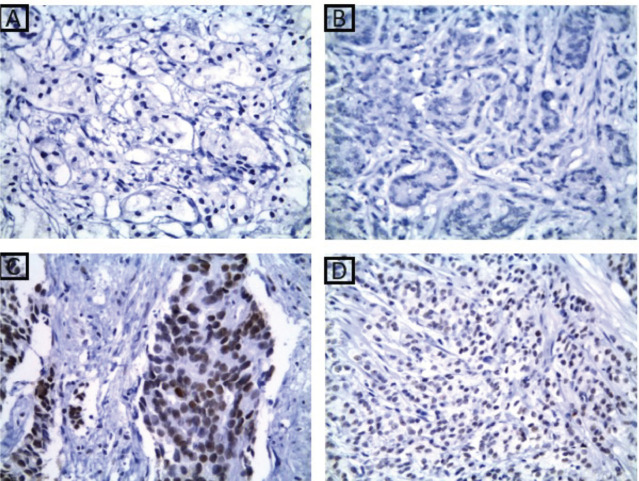
MLH1 immunohistochemical results. (a): A case of well-differentiated PC Gleason score 3 showing absent nuclear expression of MLH1 ×200. (b): A case of moderately differentiated PC Gleason score 7 showing absent nuclear expression of MLH1 ×400. (c): A case of moderately differentiated PC Gleason score 7 showing retained nuclear expression of MLH1 ×400. (d): A case of poorly differentiated PC Gleason score 9 showing retained nuclear expression of MLH1 ×400.

At the current study, MSH2 and MLH1 immunoexpression was absent in 36% and 38% of cases, respectively, as shown in Table 2 and Figures 2 and 3. Regarding the relation between each marker and the studied clinicopathological features and histopathological results, it is demonstrated in Tables 1–6. There was a significant positive relation between MSH2 immunoexpression and both PSA serum level and P53 serum concentration (*p* value 0.001*). Also, there was a significant relation between MSH2 immunoexpression and tumour size, nodal metastasis, distant metastasis and grade grouping. While MLH1 immunoexpression showed a significant relation with human P53 serum concentrations only (*p* value 0.035*), as shown in Table 5. Moreover, MLH1 immunoexpression showed only significant relation with nodal metastasis and tumour burden, *p* value was 0.033* and 0.001*, respectively.

### P53 protein immunoexpression results

**Figure 4.**
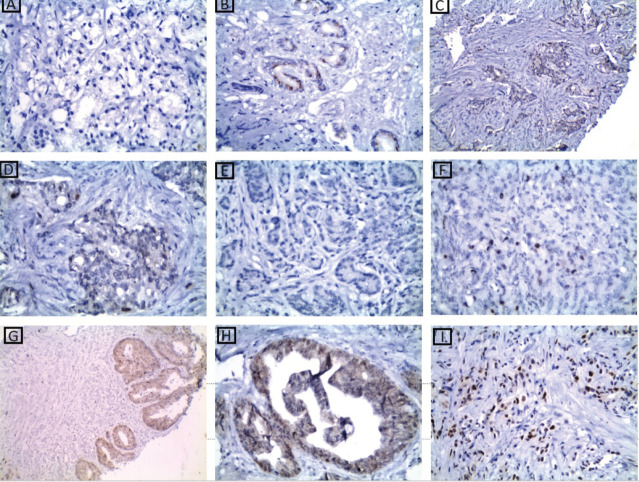
p53 immunohistochemical results. (a): A case of well-differentiated PC Gleason score 3 showing low nuclear expression of p53 ×200. (b): A case of moderately differentiated PC Gleason score 7 showing low nuclear expression of p53 ×400. (c and d): A case of moderately differentiated PC Gleason score 7 showing low nuclear expression of p53 ×200 and ×400, respectively. (e): A case of moderately differentiated PC Gleason score 7 showing low nuclear expression of p53 ×400. (f): A case of poorly differentiated PC Gleason score 9 showing low nuclear expression of p53 ×400. (g and h): A case of moderately differentiated PC Gleason score 7 showing high nuclear expression of p53 ×200 ×400, respectively. (i): A case of poorly differentiated PC Gleason score 9 showing high nuclear expression of p53 ×400.

Tumour P53 expression was found to be low in 60% of studied prostatic carcinoma (PC) cases as shown in Table 7 and Figure 4. Regarding the relation between P53 immunoexpression and the available clinicopathological features, there was a significant positive relation between its expression and serum PSA levels and serum Human P53 concentrations (*p* value 0.001*) for both, as illustrated in Table 8. There was no statistically significant relation between P53 immunoexpression and any of the studied histopathological results as shown in Table 9.

## Discussion

PCa ranks as the second most prevalent cancer among men and represents the fifth leading cause of cancer-related deaths worldwide. The wide spectrum of biological behaviour exhibited by prostatic neoplasms poses a difficult problem in predicting the clinical course for the individual patient. Histopathology together with the specified pathological markers plays a very important role in its preoperative and postoperative evaluation. Although PCa are slowly growing tumours, they vary widely in their aggressiveness [2].

Many traditional prognostic factors are present, such as tumour grade, clinical stage and pre-treatment PSA plasma levels, but they are of limited prognostic value for individual patient. So, the need for new prognostic molecular factors is necessary [11].

In recent years, some cases with PC have been linked with defects in MMR proteins. Worldwide, the detection of MMR proteins by IHC is routinely done in colorectal and endometrial adenocarcinoma. In a study by Hashemi *et al* [35] and another by Qasim *et al* [20], a high frequency of MMR deficiency in colorectal and endometrial adenocarcinoma was observed, respectively [20], but it is quite rare in PCa and its histology and molecular morphology were incompletely described. Thus, this study is trying to identify the features of PC with MSI [33].

In a study by Rodrigues *et al* [23], they revealed that impairment of MMR genes has been linked to the risk of PCa in men with Lynch syndrome. Moreover, it has been recently studied in sporadic cases of PCa and it has indeed been associated with worse patient outcomes, while favourable response to immune checkpoint blockade in those with MMR deficiency has also been reported. However, the clinical impact of MMR deficiency, especially that detected by IHC, in patients with PCa remains largely unknown [24].

In the current study, IHC expression of MSH2 and MLH1 was evaluated, in terms of their deficiency in prostatic adenocarcinoma, as they are known to play an important role in carcinogenesis, via MSI, particularly defects within the MSH2 gene. So, it provides valuable insights into the genetic characteristics of these tumours and help us identifying patients who may benefit from immunotherapy. Also, analysis of their relationship with respect to age, Gleason score, tumour size, nodal metastasis, tumour burden, grade group, PSA and P53 levels were done to assess their predictive role in predicting aggressive behaviour of PCa.

At the present study, MSH2 and MLH1 immunoexpression were absent in 36% and 38% of cases, respectively. Also, there was a significant positive relation between MSH2 immunoexpression and both PSA serum level and p53 serum concentrations. Also, there was a significant relation between MSH2 immunoexpression and tumour size, nodal metastasis, distant metastasis and grade grouping but MSH1 immunoexpression showed significant relations with only P53 serum concentration, tumour metastasis and tumour burden. Loss of at least one of the

MMR proteins was identified in 50 (22.7%) cases. These findings indicate a strong association between the deficiency of MMR proteins and biological and aggressive behaviours of invasive PCa.

A few immunohistochemical studies have reported the incidence of MMR deficiency in PCa. Sharma *et al* [26]reported that MLH1, MSH2, MSH6 and PMS2 were lost in 2 (0.9%), 6 (2.7%), 37 (16.8%) and 27 (12.3%) of PCa, respectively. They explained these low incidences by some limitations as using TMA consisting of only 1-mm tissue cores that may not be representative of the invasive cancer in each case, which may thus produce false-negative results.

According to Sharma *et al* [26], there were no statistically significant associations between MMR deficiency and patient age, family history of PCa, Gleason score or pT/pN stage. However, the levels of preoperative PSA were significantly (*p*  =  0.015) higher in patients with MMR deficiency (mean ±  SD: 9.12  ±  9.01  ng/mL) than in those without abnormal MMR (5.76  ±  3.17  ng/mL).

Among other studies, Guedes *et al* [8, 9] demonstrated that MSH2 loss was significantly more often seen in tumours with Gleason score 9–10/Grade Group 5 than in those with Gleason score ≤8/Grade Group ≤4. In addition, Wilczak *et al* [31] noted elevated expression of MLH1, MSH6 and PMS2 in PCa, which was associated with higher Gleason score or pT stage, LN metastasis or earlier biochemical recurrence. They concluded that high expression of MMR genes was associated with signs of genomic instability. So, as the number of deletions increased, the proportion of cancers showing strong MMR gene expression increased.

In accordance with previous observations and results, our research showed concurrent loss of MLH1 and MSH2. Moreover, the levels of preoperative PSA in patients with MMR deficiency were significantly higher than those without MMR deficiency. These results were recently confirmed by a study conducted by Grindedal *et al* [7] who found statistically significant increased incidence of prostatic cancer among MSH2 and MSH6 carriers, where the standardized incidence ratio was as high as 13 and 13.74, respectively, which also showed aggressive behaviour. Moreover, they concluded that their findings support continued PSA screening of MSH2 and MSH6 carriers.

TP53 inactivation is the genomic biomarker most consistently associated with adverse outcomes in primary and metastatic PCa. Many of the pathogenic TP53 mutations in PCa (70%) are protein-stabilising missense mutations that lead to nuclear accumulation. Other TP53 alterations are truncating mutations (or rarely deep deletions) leading to protein loss [28].

Although multiple studies have shown an association between p53 expression and TP53 mutation, inconsistencies were noted by others. These discrepancies could be attributed to limitations of the IHC assay, including antibodies used for detection or to study cohort selection [8, 9]. But, Gesztes *et al* [4] confirmed the association between pathogenic TP53 mutations and higher p53 expression, which supports the usage of IHC staining of p53 as a substitute for detecting TP53 mutations.

The focality of TP53 alterations in primary PCa can lead to differences in IHC interpretations or DNA sequencing assays. Since p53 IHC detection depends on the increased half-life of the mutant proteins, proteins with destabilising mutations may escape detection. The lower frequency of TP53 mutations in localised PCa could reduce the likelihood of finding an association with increased p53 expression. Since p53 nuclear accumulation is far more frequent in higher grade carcinomas, performing IHC on all primary PCa at diagnosis is unlikely to establish the expected association [10].

According to the current work, tumour P53 expression was found to be low in 60% of the studied PC cases. These findings are quite similar to those reported by Gesztes *et al* [4] who found that nearly half (49.8%) of the examined prostatic tumours showed focal p53 expression. But, Shurbaji *et al* [27] reported that immunoreactivity for p53 was only seen in 21% of their studied cancer cases. This discrepancy was documented in Rejeb *et al* [22] who showed widely variable rate of p53 expression ranging from 1.1% in localised tumours to 54.7% in surgically treated PCa which may be attributed to differences in the methodology of p53 immunostaining assessment, the differences in the techniques, studied specimens (one section or multiple cores of tumour tissue) and patient’s stage (primary tumours, metastasis).

In the present study, p53 expression was significantly positively correlated with serum PSA level, but no statistically significant relation was found between its expression and any of the studied clinicopathological variables or histopathological findings, which could be related to the small sample size. On the contrary, there was statistically significant relation between P53 expression and MSH2 expression denoting its association with high-grade tumours and poor prognosis.

Gesztes *et al* [4] also found that high p53 expression was significantly associated with distant metastasis and lymph vascular invasion. Moreover, Shurbaji *et al* [27] reported that immunoreactivity for p53 was strongly related to progression of PCa in the form of grading and staging denoting that inactivation of p53 is a late event during PCa progression.

## Conclusion

MMR protein loss, especially MSH2, was seen in a significant subset of PCa. Interestingly, it was associated with significantly higher levels of serum PSA and p53. Moreover, it may be associated with poor prognostic features as large tumour size, higher grade grouping and finally nodal and distant metastasis.

## Conflicts of interest

The authors did not have any conflicts of interest.

## Funding

The authors declare that they have no fundings.

## Author contributions

First and corresponding author: the idea of the research, protocol writing, data collection and paper writing and revision.

Second author: idea participation, providing clinical data cases and writing their results.

Third author: performing serological tests, writing their results and statistical analyses of their data.

Fourth author: idea participation, data collection, writing the background and discussion.

## Figures and Tables

**Table 1.** Clinicopathological features of studied cases.

Clinicopathological features		Range	Mean ± SD
Age		52–87	66.52 ± 8.24
PSA		0.50–100	32.41 ± 35.44
Human p53 concentration		0.78–50	21.34 ± 16.21
**Clinical staging**		***N***	**%**
T	T 1c	16	32
	T 2	16	32
	T 3	7	14
	T 4	11	22
N	N 0	28	56
	N 1	22	44
M	M 0	43	86
	M 1	7	14
Pathological findings		***N***	**%**
Perineural invasion	No	28	56
	Yes	22	44
Gleason score	2	5	10
	3	4	8
	4	6	12
	5	5	10
	6	7	14
	7	7	14
	8	7	14
	9	6	12
	10	3	6
Grade group	Grade group I	27	54
	Grade group II	3	6
	Grade group III	4	8
	Grade group IV	7	14
	Grade group V	9	18
Tumor burden	0	25	50
	1	25	50

**Table 2.** MMR proteins immunoexpression results.

Immunomarker	Result	No	Percent
MSH2	Absent	18	36
	Present	32	64
MLH1	Absent	19	38
	Present	31	62

**Table 3.** Relation between MSH2 immunoexpression and clinicopathological features.

MSH 2		Range			Mean	±	S. D	*t*. test	*p*. value
Age	Absent	55	–	87	67.50	±	8.81	0.626	0.534
	Present	52	–	80	65.97	±	8.00		
PSA	Absent	3	–	100	61.29	±	34.77	5.439	0.001*
	Present	0.5	–	95	16.16	±	23.77		
Human p53 serum concentration	Absent	4.5	–	50	34.88	±	15.76	5.660	0.001*
	Present	0.78	–	42	13.72	±	10.63		

* *p* values of less than 0.05 were considered statistically significant

**Table 4.** Relation between MSH2 immunoexpression, clinical staging and histopathological features.

			MSH 2		*X* ^2^	*p*-value
			No	Yes		
T	T 1c	*N*	0	16	16.000	0.001*
		%	0.0%	50.0%		
	T 2	*N*	7	9		
		%	38.9%	28.1%		
	T 3	*N*	3	4		
		%	16.7%	12.5%		
	T 4	*N*	8	3		
		%	44.4%	9.4%		
N	N 0	*N*	3	25	17.659	0.001*
		%	16.7%	78.1%		
	N 1	*N*	15	7		
		%	83.3%	21.9%		
M	M 0	*N*	13	30	4.434	0.035*
		%	72.2%	93.8%		
	M 1	*N*	5	2		
		%	27.8%	6.3%		
P 53	Low	*N*	5	25	12.167	0.001*
		%	27.8%	78.1%		
	High	*N*	13	7		
		%	72.2%	21.9%		
Perineural invasion	No	*N*	7	21	3.342	0.068
		%	38.9%	65.6%		
	Yes	*N*	11	11		
		%	61.1%	34.4%		
Grade group	Grade I	N	6	21	12.660	0.013*
		%	33.3%	65.6%		
	Grade II	*N*	1	2		
		%	5.6%	6.3%		
	Grade III	*N*	0	4		
		%	0.0%	12.5%		
	Grade IV	*N*	4	3		
		%	22.2%	9.4%		
	Grade V	*N*	7	2		
		%	38.9%	6.3%		
Tumor burden	0	*N*	7	18	1.389	0.239
		%	38.9%	56.3%		
	1	*N*	11	14		
		%	61.1%	43.8%		

* *p* values of less than 0.05 were considered statistically significant

**Table 5.** Relation between MLH1 immunoexpression and clinicopathological features.

MLH 1		Range			Mean	±	S. D	*t*. test	*p*. value
Age	No	53	–	87	65.74	±	8.80	0.522	0.604
	Yes	52	–	80	67.00	±	8.00		
PSA	No	0.5	–	100	41.98	±	35.50	1.514	0.136
	Yes	0.5	–	100	26.55	±	34.66		
Human p53 serum concentration	No	4.5	–	50	27.48	±	16.49	2.175	0.035*
	Yes	0.78	–	50	17.57	±	15.09		

* *p* values of less than 0.05 were considered statistically significant

**Table 6.** Relation between MLH1 immunoexpression, clinical staging and histopathological features.

			MLH 1		*X* ^2^	*p*-value
			No	Yes		
T	T 1c	***N***	3	13	4.885	0.180
		%	15.8%	41.9%		
	T 2	*N*	6	10		
		%	31.6%	32.3%		
	T 3	*N*	4	3		
		%	21.1%	9.7%		
	T 4	*N*	6	5		
		%	31.6%	16.1%		
N	N 0	N	7	21	4.565	0.033*
		%	36.8%	67.7%		
	N 1	*N*	12	10		
		%	63.2%	32.3%		
M	M 0	*N*	15	28	1.266	0.261
		%	78.9%	90.3%		
	M 1	*N*	4	3		
		%	21.1%	9.7%		
P 53	Low	*N*	9	21	2.037	0.153
		%	47.4%	67.7%		
	High	*N*	10	10		
		%	52.6%	32.3%		
Perineural invasion	No	*N*	8	20	2.401	0.121
		%	42.1%	64.5%		
	Yes	*N*	11	11		
		%	57.9%	35.5%		
Grade	Grade I	*N*	9	18	4.995	0.288
		%	47.4%	58.1%		
	Grade II	*N*	1	2		
		%	5.3%	6.5%		
	Grade III	*N*	0	4		
		%	0.0%	12.9%		
	Grade IV	*N*	4	3		
		%	21.1%	9.7%		
	Grade V	*N*	5	4		
		%	26.3%	12.9%		
Tumor burden	0	*N*	7	18	18.889	0.001*
		%	36.8%	58.1%		
	1	*N*	12	13		
		%	63.2%	41.9%		

* *p* values of less than 0.05 were considered statistically significant

**Table 7.** P53 immunoexpression in studied cases.

Immunomarker	Result	No	Percent
**P 53**	Low	30	60
	High	20	40

**Table 8.** Relation between P53 immunoexpression and clinicopathological features.

P 53		Range			Mean	±	S. D	*t*. test	*p*. value
Age	Low	52	–	87	67.13	±	7.97	0.640	0.525
	High	53	–	80	65.60	±	8.77		
PSA	Low	0.5	–	50	13.45	±	13.58	6.118	0.001*
	High	2	–	100	60.85	±	39.22		
Human p53 serum concentration	Low	0.78	–	25	10.40	±	6.41	10.499	0.001*
	High	12.5	–	50	37.75	±	11.96		

* *p* values of less than 0.05 were considered statistically significant

**Table 9.** Relation between P53 immunoexpression, clinical staging and histopathological results.

			P 53		*X* ^2^	*p*-value
			Low	High		
T	T 1c	*N*	13	3	5.689	0.128
		%	43.3%	15.0%		
	T 2	*N*	9	7		
		%	30.0%	35.0%		
	T 3	*N*	4	3		
		%	13.3%	15.0%		
	T 4	*N*	4	7		
		%	13.3%	35.0%		
N	N 0	*N*	20	8	3.463	0.063
		%	66.7%	40.0%		
	N 1	*N*	10	12		
		%	33.3%	60.0%		
M	M 0	*N*	27	16	0.997	0.318
		%	90.0%	80.0%		
	M 1	*N*	3	4		
		%	10.0%	20.0%		
Perineural invasion	No	*N*	17	11	0.014	0.907
		%	56.7%	55.0%		
	Yes	*N*	13	9		
		%	43.3%	45.0%		
Grade	Grade I	*N*	18	9	5.820	0.213
		%	60.0%	45.0%		
	Grade II	*N*	1	2		
		%	3.3%	10.0%		
	Grade III	*N*	4	0		
		%	13.3%	0.0%		
	Grade IV	*N*	3	4		
		%	10.0%	20.0%		
	Grade V	*N*	4	5		
		%	13.3%	25.0%		
Tumor burden	0	*N*	17	8	1.333	0.248
		%	56.7%	40.0%		
	1	*N*	13	12		
		%	43.3%	60.0%		

*Relation between MMR proteins and P53 immunoexpression*

There was a statistically significant relation between P53 expression and MSH2 expression (*p* value 0.001*), but not with MLH1 immunosuppression (*p* value 0.153), as shown in Table 10.

**Table 10.** Relation between P53 expression and MMR proteins immunoexpression.

			MSH2		*X* ^2^	*p*-value
			Yes	no		
P 53	Low	*N*	5	25	12.167	0.001*
		%	27.8%	78.1%		
	High	*N*	13	7		
		%	72.2%	21.9%		
MLH1						
P 53	Low	*N*	9	21	2.037	0.153
		%	47.4%	67.7%		
	High	*N*	10	10		
		%	52.6%	32.3%		

* *p* values of less than 0.05 were considered statistically significant
